# Aged glomeruli: a link between PD-1 and podocytes

**DOI:** 10.1172/JCI162330

**Published:** 2022-08-15

**Authors:** Samuel Mon-Wei Yu, John Cijiang He

**Affiliations:** 1 Division of Nephrology, Department of Medicine, and; 2 Department of Pharmacological Sciences, Mount Sinai School of Medicine, New York, New York, USA.; 3 James J. Peters Veteran Administration Medical Center, New York, New York, USA.

## Abstract

Understanding the loss of kidney function resulting from kidney aging has become an emerging research focus that will facilitate the future development of antisenolytic treatments. In this issue of the *JCI*, Pippin et al. first identified PD-1 upregulation in the aged mouse podocyte via unbiased RNA-seq analysis. Overexpression of PD-1 in immortalized mouse podocytes induced cell death and a senescence-associated secretory phenotype, suggesting the pathological role of PD-1 upregulation in aged podocytes. Blocking PD-1 signaling via a neutralizing anti-PD-1 antibody reversed the aged phenotype in the aged mice and ameliorated proteinuria in an experimental focal segmental glomerulosclerosis (FSGS) mouse model. These findings highlight the role of PD-1 signaling in kidney aging and its therapeutic potential for human clinical trials.

## How kidney aging relates to podocytes and PD-1

According to the latest data from the WHO, while our life expectancy continues to increase in the past decade, healthy-adjusted life expectancy without disability has not proportionally increased (1). These data highlight an important and unmet need to mitigate disabilities in the elderly population to improve their quality of life. For example, multiple studies have demonstrated a higher prevalence of chronic kidney disease (CKD) among the elderly population (2, 3), which could result in physical impairment, cognitive dysfunction, and frailty (4). CKD development in older individuals is partly due to the natural loss of glomerular filtration rate (GFR) — so-called “kidney aging,” — and and, frequently, concomitant comorbidities, such as cardiovascular disease and diabetes, could hasten the disease process. Although treatments such as sodium-glucose cotransporter 2 inhibitor have shown impressive amelioration of diabetic kidney disease and CKD progression (5), therapeutics targeted against kidney aging have remained relatively sparse.

**Figure 1:**
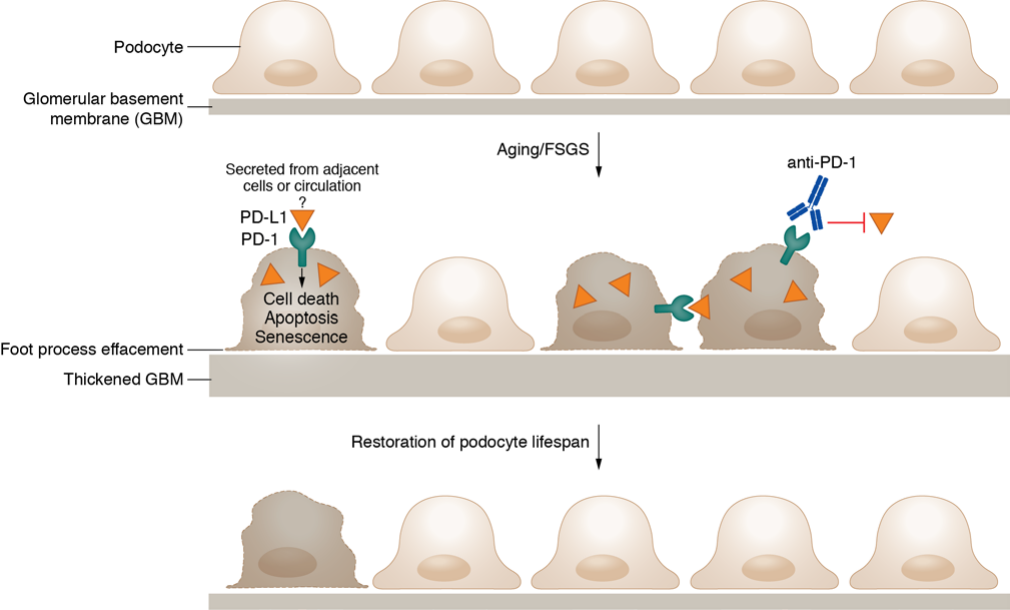
PD-1 signaling is involved with cellular senescence and loss in the aged or injured mouse podocytes. Upregulation of PD-1 expression in aged podocytes, or those from mice with FSGS, leads to a series of detrimental effects, such as cell death, increased cellular senescence, and finally, podocyte loss. Conversely, inhibiting PD-1 signaling via a PD-1-specific antibody improves podocyte ultrastructure, function, gene expression, cell density, and lifespan.

Compared to other kidney resident cell populations such as kidney tubules, podocytes are generally considered a terminally differentiated cell population. Podocyte loss is not only associated with de novo glomerular diseases but also with kidney aging seen in both murine and human kidney biopsies (6). After the loss or detachment of podocytes, the remaining podocytes undergo compensatory changes with hypertrophy, but their functional capacity is substantially reduced. Given that podocyte loss becomes more and more irreversible with age, understanding the underlying mechanisms could facilitate future drug targets. In this issue of the *JCI*, Pippin et al. (7) extrapolated from their previous work — in which the authors isolated podocytes from young versus aged mice followed by bulk RNA-seq analysis — and identified an increase in gene expression, including programmed cell death 1 (PD-1) and its cognate ligands programmed cell death 1 ligand 1 and 2 (PD-L1, PD-L2) (8). PD-1 and its ligands have garnered great attention in the past years, largely due to the clinical use of PD-1/PD-L1 inhibitors (also known as immune checkpoint inhibitors) in various cancer treatments. Robustly expressed in the activated T cells, PD-1 engages with its ligands, presented on antigen-presenting cells or cancer cells, to dampen adaptive immune responses (9). In the kidneys, tubular expression of PD-L1 has been shown in vitro and in vivo, and upregulation of PD-L1 on tubular cells may attenuate acute T cell-mediated rejection (10). Therefore, it is intuitive to assume that the expression of PD-L1 in the kidneys is also immunosuppressive and renoprotective in the context of kidney inflammation or transplant. Based on an unbiased analysis, Pippin and colleagues generated an alternative hypothesis that upregulation of PD-1 in aged podocytes could, in fact, contribute to the aging process and podocyte loss (Figure 1) (7).

## The effects of PD-1 blockade on glomerular health

Pippin and colleagues first confirmed their findings of PD-1/PD-L1 upregulation by staining young and aged mouse and human kidneys, in which increased PD-1 was observed in aged podocytes, parietal epithelial cells, and LTL^+^-tubular cells, but not in mesangial cells or glomerular endothelial cells (GENs). These data were further strengthened by the association between increased human glomerular PD-1 gene transcripts, older age, and reduced eGFR. Then, using immortalized mouse podocytes as an in vitro system, the authors proved that overexpression of PD-1 led to increased cell death, which could be inhibited by a neutralizing anti-PD-1 antibody (aPD1ab) and a caspase-3 inhibitor. The authors went on to test the effects of PD-1 blockade on clinical outcomes by injecting aPD1ab or the control IgG2a antibody into aged mice weekly. After 8 weeks, aged mice receiving aPD1ab had lessened aging-related markers, such as senescence-associated beta-galactosidase (SA-β-gal), p16, and p19 in the glomeruli, as well as a higher podocyte density, compared with age-matched controls. Considering the intraglomerular cell-cell crosstalk, the authors further examined parietal epithelial cells (PECs) and GENs. They found that, despite no notable changes in the cell numbers, both PECs and GENs demonstrated reduced expression of different activation markers indicative of a healthier phenotype. mRNA-seq was performed on podocytes isolated from these injected mice and revealed a series of restored canonical podocyte genes associated with healthy podocytes, such as nephrin (*Nphs1*) and vascular endothelial growth factor A (*Vegfa*) in the aPD1ab group. Injection with the aPD1ab also increased genes regulating oxidative phosphorylation and glycolysis, suggesting improved cellular metabolism. On the contrary, genes related to cell death and inflammation were reduced in the aPD1ab group, further confirmed by immunohistochemistry. Interestingly, the authors similarly observed decreased tubular kidney injury molecule-1 (KIM-1) staining in aPD1ab treated aged mice. Although there was no difference in serum blood urea nitrogen (BUN) between aPD1ab and the control group, the renoprotective effects may expand beyond podocytes to tubules (7).

Focal segmental glomerulosclerosis (FSGS) is commonly seen in aged kidneys, likely due to increasing podocyte loss and hyperfiltration (11). Pippin and colleagues next tested if aPD1ab could also improve the outcome of FSGS. First, the authors confirmed that in both human and mouse FSGS models, there was an upregulation of PD-1 expression similar to that seen in aged kidneys. Then, after injecting sheep anti-glomerular antibody as an experimental FSGS model, the authors administered 4 doses of aPD1ab or IgG2a control and analyzed the functional outcome on day 14. Proteinuria was reduced in the aPD1ab treated mice, whereas other functional tests, such as serum soluble urokinase Plasminogen Activator Receptor and staining of Nphs1 on podocytes, remained unchanged. These studies further corroborated the protective effects of PD-1 block observed in the podocytes of aged mice (7).

## Unanswered questions and clinical correlation

Pippin et al. provide a link between PD-1 expression and kidney aging and demonstrate great therapeutic potential in preserving glomerular health by PD-1 blockade in mice (7). Although PD-1/PD-L1 inhibitors are known to cause acute kidney injury (AKI) (12), the current data provide strong evidence and underlying mechanisms suggesting that these inhibitors might lessen podocyte injury in aged kidneys and FSGS. The study also suggests that an inhibitor of PD-1/PD-L1 could serve as a senolytic agent to clear senescent cells. Since kidney cell senescence plays a major role in renal fibrosis (13), these agents have the potential to treat podocyte disorders and also be used as an antifibrosis therapy.

Pippin and colleagues provided a thoughtful discussion on the limitations of the study (7). Nevertheless, there are other potential interpretations regarding the underlying renoprotective mechanisms observed in the aPD1ab-treated mice. First, since PD-1 is a receptor, the activation of PD-1 signaling requires its cognate ligands (PD-L1/PD-L2) to initiate downstream signaling. Although there was an upregulation of PD-L1/PD-L2 from podocytes of aged mice compared to young mice, it is yet to be determined if PD-1 signaling in the podocytes is triggered by a local paracrine or autocrine signal, or by circulating soluble PD-L1 produced by immune cells (14). PD-1 is also expressed on myeloid cells, and previous data suggested that the myeloid cell-specific deletion of PD-1 or anti-PD-1 antibody induces monocytic lineage differentiation into macrophages (CD11b^+^F4/80^+^) (15). Since myeloid cells are implicated in the pathogenesis of proteinuric kidney disease (16) and FSGS (17), it would also be of interest to characterize the immune phenotypes of the mice injected with aPD1ab versus the control antibody. Second, previous studies have shown that, while podocyte dysfunction can cause tubular injury, tubular dysfunction can, indeed, affect podocyte health reciprocally (18). Since the authors demonstrated both an improvement of podocyte function and a reduction of tubular KIM-1 expression in the aged mice treated with aPD1ab, generating podocyte- and tubular-specific PD-1 knockout mice would be useful to verify the cell type-specific role of PD-1. Lastly, PD-1 is used as a surface marker to detect T cell exhaustion, as are mucin domain-containing protein 3 (TIM3), and Lymphocyte Activating 3 (LAG3). It might be possible that podocytes (and even tubules) upregulate PD-1 via an unknown mechanism in response to injury, and PD-1 expression could similarly be a surrogate to detect aged podocytes.

A trial using PD-1 inhibitors in patients with FSGS could be considered from the clinical perspective. However, it might be challenging to design such a trial in patients with aged kidney disease, given that the markers to detect cellular senescence and aging are still under debate (19). Furthermore, nephrotoxicity from PD-1 inhibitors should also be considered in human clinical studies, particularly in the elderly population with higher risks of AKI, among other comorbidities. Finally, it would be intriguing to evaluate the possible renoprotective effects of PD-1 blockade on AKI to CKD transition and kidney fibrosis. In summary, the present study by Pippin et al. (7) introduces PD1 signaling as a player involved in podocyte aging and brings hope to the treatment of kidney aging with an exciting therapeutic approach.

## Version 1 08/15/2022

Electronic publication

## References

[1] https://cdn.who.int/media/docs/default-source/gho-documents/global-health-estimates/ghe2019_life-table-methods.pdf?sfvrsn=c433c229_5.

[2] https://www.niddk.nih.gov/about-niddk/strategic-plans-reports/us-renal-data-system-report.

[3] Denic A (2016). Structural and functional changes with the aging kidney. Adv Chronic Kidney Dis.

[4] Anand S (2014). Aging and chronic kidney disease: the impact on physical function and cognition. J Gerontol A Biol Sci Med Sci.

[5] DeFronzo RA (2021). Pathophysiology of diabetic kidney disease: impact of SGLT2 inhibitors. Nat Rev Nephrol.

[6] Shankland SJ (2021). Podocyte aging: why and how getting old matters. J Am Soc Nephrol.

[7] Pippin JW (2022). Upregulated PD-1 signaling antagonizes glomerular health in aged kidneys and disease. J Clin Invest.

[8] Wang Y (2020). Global transcriptomic changes occur in aged mouse podocytes. Kidney Int.

[9] Sharpe AH, Pauken KE (2018). The diverse functions of the PD1 inhibitory pathway. Nat Rev Immunol.

[10] Starke A (2010). Renal tubular PD-L1 (CD274) suppresses alloreactive human T-cell responses. Kidney Int.

[11] De Vriese AS (2018). Differentiating primary, genetic, and secondary FSGS in adults: a clinicopathologic approach. J Am Soc Nephrol.

[12] Gupta S (2020). Immune checkpoint inhibitor nephrotoxicity: update 2020. Kidney360.

[13] Docherty MH (2019). Cellular senescence in the kidney. J Am Soc Nephrol.

[14] Patsoukis N (2020). Revisiting the PD-1 pathway. Sci Adv.

[15] Strauss L (2020). Targeted deletion of PD-1 in myeloid cells induces antitumor immunity. Sci Immunol.

[16] Hahm E (2017). Bone marrow-derived immature myeloid cells are a main source of circulating suPAR contributing to proteinuric kidney disease. Nat Med.

[17] Latt KZ (2022). Urine single-cell RNA sequencing in focal segmental glomerulosclerosis reveals inflammatory signatures. Kidney Int Rep.

[18] Yu SM, Bonventre JV (2018). Acute kidney injury and progression of diabetic kidney disease. Adv Chronic Kidney Dis.

[19] He S, Sharpless NE (2017). Senescence in health and disease. Cell.

